# Effect of a controlled release device containing minocycline microspheres on the treatment of chronic periodontitis: A comparative study

**DOI:** 10.4103/0972-124X.55844

**Published:** 2009

**Authors:** V. Gopinath, T. Ramakrishnan, Pamela Emmadi, N. Ambalavanan, Biju Mammen, Vijayalakshmi

**Affiliations:** *Senior Lecturer, Department of Periodontics, Saveetha Dental College and Hospital, Poonamalee High Road, Velapanchavadi, Chennai - 77, India*; 1 *Professor, Meenakshi Ammal Dental College and Hospital, Alapakkam Main Road, Maduravoyal, Chennai - 95, India*; 2 *Professor and Head, Meenakshi Ammal Dental College and Hospital, Alapakkam Main Road, Maduravoyal, Chennai - 95, India*; 3 *Associate Professor, Meenakshi Ammal Dental College and Hospital, Alapakkam Main Road, Maduravoyal, Chennai - 95, India*; 4 *Lecturer, Meenakshi Ammal Dental College and Hospital, Alapakkam Main Road, Maduravoyal, Chennai - 95, India*

**Keywords:** Antimicrobial agents, local drug delivery systems, minocycline microspheres (Arestin™), periodontitis

## Abstract

**Introduction:**

Adjunctive therapy with locally delivered antimicrobials has resulted in improved clinical outcomes. The aim of this study was to evaluate the efficacy and safety of locally administered minocycline microspheres (Arestin™) in the treatment of chronic periodontitis.

**Materials and Methods:**

A total of 60 sites from 15 patients in the age group of 35-50 years, who had periodontal pockets measuring 5-8 mm and had been diagnosed with chronic periodontitis, were selected for the study. The selected groups were randomly assigned to either the control group (group A) or the treatment/test group (group B). Only scaling and root planing were done at the base line visit for the control sites followed by local application of Arestin™ (1 mg). Clinical parameters such as plaque index, gingival index, and gingival bleeding index were recorded at baseline, day 30, day 90, and day 180 in the selected sites of both the groups. Probing pocket depth also was recorded at baseline, day 90, and day 180 for both the groups.

**Results:**

A statistically significant reduction was observed in both groups. Group B showed better results than Group A and these differences were statistically significant.

**Conclusion:**

The results of this study clearly indicate that treatment with scaling and root planing plus minocycline microspheres (Arestin™) is more effective and safer than scaling and root planing alone in reducing the signs of chronic periodontitis.

## INTRODUCTION

Periodontal diseases are polymicrobial in nature and the complex interaction among many microbes makes the disease a challenging one to understand and treat. Host inflammatory response to plaque microorganisms causes tissue damage, leading to the destruction of the periodontal tissues in an episodic manner. Bacterial products have the ability to stimulate host cells to secrete a wide variety of inflammatory mediators that have numerous biological activities, some of which cause soft tissue and bone destruction. Cytokines such as tumor necrosis factor- α (TNF- α), interleukin-1β (IL-1β), and inflammatory mediators such as prostaglandin E_2_ are known inducers of bone resorption. These are found in high levels in gingival tissues and gingival crevicular fluid of patients with periodontal disease.[1]

The extent and degree of periodontal destruction varies widely from patient to patient, and in different sites within the same patient. These variations could be due to the presence of complex subgingival populations of microorganisms in different sites. Periodontal diseases are characterized by the presence of periodontal pockets, which are not easily accessible for plaque removal.[2]

Bacteria in the subgingival area are organized in a complex microbial biofilm. Biofilms are matrix-enclosed bacterial populations that are adherent to each other and/or surfaces or interfaces. These microbial plaques are extraordinarily persistent, difficult to eliminate, and play a vital role in periodontal disease.[3] Physical disruption of microbial plaque through hand or powered instrumentation is an effective way of eliminating biofilms. However, reinfection of periodontal pockets from recolonization of putative bacteria can occur within 60 days after mechanical therapy.[4]

Successful treatment is dependent on halting tissue destruction through the elimination or control of etiological agents, together with microbial shift towards one typically present in health. Mechanical therapy alone may fail to eliminate invasive, pathogenic bacteria because of their location within the gingiva and dental tissues, which are inaccessible to periodontal instruments. Treatment strategies aiming primarily at suppressing or eliminating specific periodontal pathogens include local and systemic administration of antibiotics.[5] In order to obtain an effective concentration of the antimicrobial drug which would reach the microorganisms in the periodontal tissues after systemic administration, repeated intake is required over a prolonged period of time.

One of the biggest benefits of any locally delivered drug is that it does not require patient compliance for regular drug intake. The clinician places the drug which releases the antimicrobial for an extended period of time at a steady pharmacological level.[6] Another advantage of a locally delivered drug is that it can be placed adjacent to disease sites in the periodontium, leaving other parts of the body unaffected.[6]

Arestin™ is made up of minocycline, a semi-derivative of tetracycline, and a very potent broad-spectrum antibiotic. Minocycline has significant antimicrobial activity against a wide range of organisms as well as an anticollagenase effect.[7] Minocycline works by interfering with protein synthesis in the bacterial cell wall.

Arestin™ delivers minocycline in a powdered microsphere delivery system. The microspheres have diameters ranging from 20 to 60 μ. The active ingredient is minocycline hydrochloride which exists as particles distributed throughout the interior of the microspheres. When Arestin™ is administered, it immediately adheres to the periodontal pocket.[6]

Gingival crevicular fluid hydrolyzes the polymer, causing water-filled channels to form inside the microspheres. These holes provide escape routes for the encapsulated antibiotic for sustained release. The active drug dissolves and diffuses out of the microspheres through the channels into the surrounding tissues. After ten days, the microspheres are fragmented and continue to release minocycline for 14 days or longer; eventually, these microspheres completely bioresorb.

Traditional therapies such as tooth brushing, flossing, subgingival irrigation, and mechanical debridement are successful for patients with mild periodontal diseases. However, as the periodontal pocket deepens, the patient's home care procedures as well as professional debridement loses effectiveness, making local drug delivery a viable option. The addition of local delivery can also help to maintain or control the disease between maintenance visits.[8]

The purpose of this study was to compare the clinical effects of scaling and root planing with those of scaling, root planing, and local administration of minocycline hydrochloride (1 mg) (Arestin™) delivered subgingivally in patients with chronic periodontitis.

## MATERIALS AND METHODS

A total number of 15 patients aged 35-50 years diagnosed with chronic periodontitis and having probing depths ranging from 5 to 8 mm as well as radiographic evidence of bone loss, were selected for the study from the Department of Periodontics, Meenakshi Ammal Dental College and Hospitals, Chennai.

Ethical approval was obtained from the Institutional ethical committee for the study.

Inclusion criteria for patient selection.

Patients in the age group of 35-50 years.

Patients diagnosed as suffering from chronic periodontitis having a probing pocket depth of 5 to 8 mm with radiographic evidence of alveolar bone loss and without mobility of teeth.

Patients willing to take part in the study and maintain appointments regularly.

Exclusion criteria for patient selection.

Patients having systemic diseases like diabetes mellitus, hypertension, bleeding disorders, hyperparathyroidism.

Pregnant women and lactating mothers.

Patients allergic to tetracyclines.

Patients who have had periodontal treatment in last six months.

Antibiotic therapy within three months prior to treatment.

Long-term therapy within a month prior to enrollment with medications that could affect periodontal status or healing.

Patients with medical or dental therapy scheduled or expected to occur during the course of this study that could have an impact on the subjects ability to complete the study.

**Figure 1:**
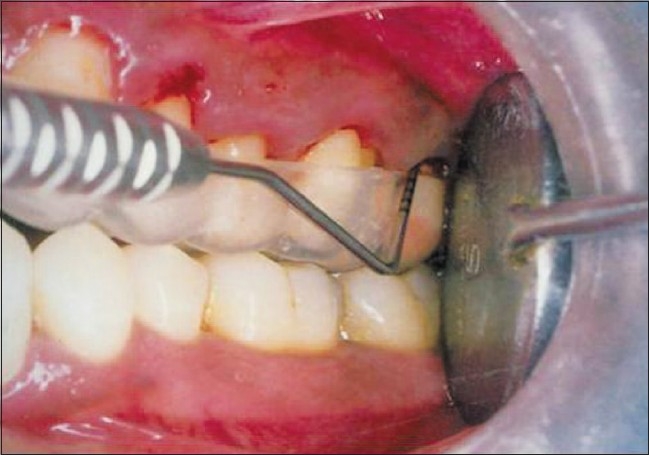
Probing with acrylic stent

A total number of 60 sites from 15 patients were selected for the study. The duration of the study was for six months. Four sites were identified for the study in each patient: Two sites served as control sites (Group A) and two sites on the contralateral side served as test sites (Group B). For all patients, general, oral and full mouth periodontal examination was carried out and informed consent was obtained from the patients. On screening day (day 0), patient evaluation was followed by impressions for the fabrication of acrylic stents required for the measurement of pocket depths in the control and test sites during the study period [Figure 1]. Variables associated were recorded on baseline day (day 0) before treatment to provide baseline data.

The following parameters were recorded:

1. Plaque index[9]
2. Gingival index[9]
3. Gingival bleeding index[9]
4. Probing pocket depth[9]

The control and test sites were grouped and treated as follows:

Group A (control) - Comprised of 30 sites; only scaling and root planing was done at the baseline visit.

Group B (test) - Comprised of 30 sites; scaling and root planing was followed by local application of Arestin™ (1 mg) at the baseline visit.

Both the control and test sites were again examined on the 30^th^ day. During this visit, all clinical parameters, except probing depth, were measured. An additional application of Arestin™ (1 mg) was given in the test sites,. The control and test sites were also examined on the 90^th^ and 180^th^ days, and all clinical parameters including probing pocket depth were recorded.

**Figure 2:**
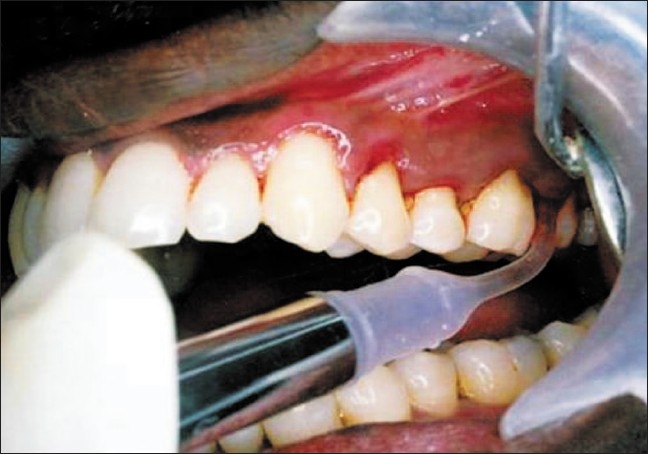
Application of minocycline microspheres

Application of minocycline microspheres [Figure 2].

After insertion of the local drug delivery system, the patients were advised not to eat hard food that could traumatize the gingiva. They were also advised not to brush the area for 12 h or to floss or use interproximal cleaning devices for ten days. During the study period, the patients were instructed to continue regular tooth brushing and interdental cleansing. They were also instructed not to use any mouth washes for the duration of the study.

### Statistical analysis

Means and standard deviations were estimated from the samples for each study group. Mean values were compared by Student's independent *t*-test/Student's paired *t*-test wherever appropriate. The proportion of positivity of the gingival bleeding index was compared between control and test groups by Pearson's chi-square test with Yate's continuity correction/Fisher's exact test (two-tailed) wherever appropriate. In the present study, *P* < 0.05 was considered as the level of significance.

## RESULTS

The mean reduction in the plaque index score in group A from day 0 to day 30 was 0.46 ± 0.43, 0.42 ± 0.42 from day 0 to day 90, and 0.53 ± 0.39 from day 0 to day 180, all statistically significant differences (*P* < 0.0001). The mean reduction in the gingival index score in group A was 0.23 ± 0.35 from day 0 to day 30, 0.28 ± 0.50 from day 0 to day 90, and 0.38 ± 0.49 from day 0 to day 180, all statistically significant differences (*P* < 0.0001). The mean reduction in probing pocket depth values for group A was 0.24 ± 1.07 from 0 to day 90, 0.37 ± 1.08 from day 0 to day 180, a difference that was not statistically significant. The mean reduction in plaque index scores in group B from day 0 to day 30 was 0.56 ± 0.45, 0.72 ± 0.32 from day 0 to day 90, and 0.78 ± 0.32 from day 0 to day 180, all statistically significant differences (*P* < 0.0001)%. The mean reduction in gingival index scores in group B was 0.72 ± 0.55 from day 0 to day 30, 0.80 ± 0.41 from day 0 to day 90, and 0.95 ± 0.49 from day 0 to day 180, all statistically significant differences (*P* < 0.0001). The mean reduction in probing pocket depth for group B was 1.73 ± 0.87 mm from 0 to day 90, and 1.66 ± 0.96 mm from day 0 to day 180, again a statistically significant difference (*P* < 0.0001) [Table 1].

**Table 1:** Changes in all variables from day 0 to different time points within group A and group B

Variable	Time point compared	Group A (control)	*P* value	Group B (test)	*P* value
				
		Change mean ± SD		Change mean ± SD	
Plaque index	Day 0 - 30	0.46 ± 0.43	< 0.0001 (sig)	0.56 ± 0.45	< 0.0001 (sig)
	Day 0 - 90	0.42 ± 0.42	< 0.0001 (sig)	0.72 ± 0.32	< 0.0001 (sig)
	Day 0 - 180	0.53 ± 0.39	< 0.0001 (sig)	0.78 ± 0.32	< 0.0001 (sig)
Gingival index	Day 0 - 30	0.23 ± 0.35	= 0.001 (sig)	0.72 ± 0.55	< 0.001 (sig)
	Day 0 - 90	0.28 ± 0.50	= 0.005 (sig)	0.80 ± 0.41	< 0.0001 (sig)
	Day 0 - 180	0.38 ± 0.49	< 0.0001 (sig)	0.95 ± 0.49	< 0.0001 (sig)
Probing pocket depth	Day 0 - 90	0.24 ± 1.07	= 0.24 (ns)	1.73 ± 0.87	< 0.0001 (sig)
	Day 0 - 180	0.37 ± 1.08	= 0.19 (ns)	1.67 ± 0.96	< 0.0001 (sig)

The mean reduction in plaque index and gingival index from 0-30^th^ day, 0-90^th^ day and 0-180^th^ day was statistically significant in both group A and group B. Mean reduction in probing pocket depth was not statistically significant in group A but was highly significant in group B

**Graph 1:**
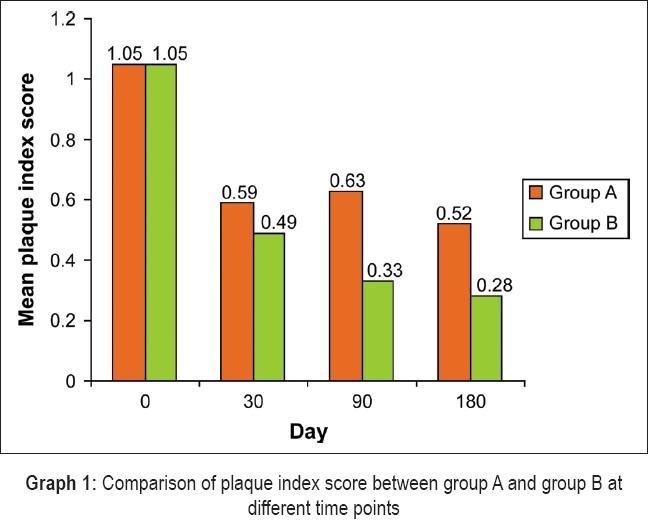
Comparison of plaque index score between group A and group B at different time points

Table 2 shows that there was no statistically significant differences in the mean plaque index scores between groups A and B on days 0 and 30. There was a statistically significant difference in the mean plaque index on days 90 and 180 between the two groups. The mean reduction in plaque index scores and the percentage of reduction were also statistically significant between days 0 and 90 and between days 0 and 180 [Graph 1].

**Table 2:** Changes in plaque index scores between group A and group B at different time points

Variable	Group A (Control) mean ± SD (*n* = 30)	Group B (Test) mean ± SD (*n* = 30)	*P* value*
Plaque index			
Day - 0	1.05 ± 0.15	1.05 ± 0.17	1.00 (ns)
Day - 30	0.59 ± 0.37	0.49 ± 0.43	0.34 (ns)
Day - 90	0.63 ± 0.38	0.33 ± 0.30	0.001 (sig)
Day - 180	0.52 ± 0.36	0.28 ± 0.30	0.007 (sig)
Change			
Day 0 - 30	0.46 ± 0.43	0.56 ± 0.45	0.38 (ns)
Day 0 - 90	0.42 ± 0.42	0.72 ± 0.32	0.003 (sig)
Day 0 - 180	0.53 ± 0.39	0.77 ± 0.32	0.01 (sig)
Percentage change from day - 0 to			
Day - 30	42.2 ± 37.6	53.0 ± 41.9	0.30 (ns)
Day - 90	38.5 ± 37.2	68.2 ± 28.2	0.001 (sig)
Day - 80	50.2 ± 34.9	74.1 ± 28.7	0.005 (sig)

Mean plaque index score in group A and group B on day 0 and 30 was not statistically significant (*P* = 1 and 0.34). On days 90 and 180, mean plaque index score difference in both groups was statistically significant (*P* = 0.001 and 0.007). There was a significant difference between the two groups in the percentage of reduction in mean plaque index score (*P* = 0.001 and 0.005) from day 0-day 90 and day 0-180.

**Graph 2:**
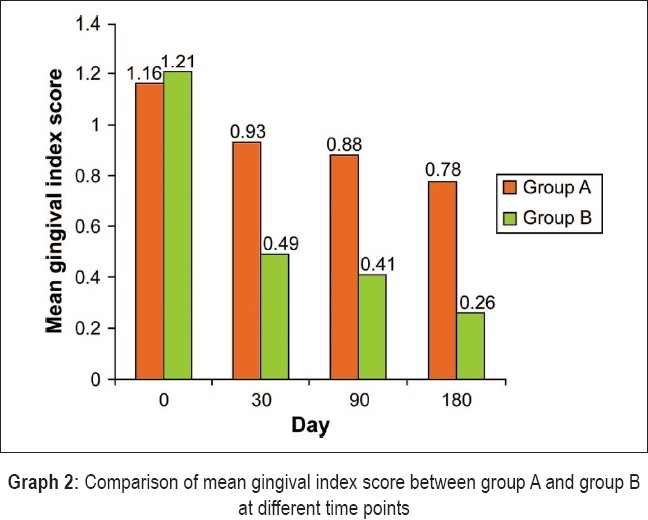
Comparison of mean gingival index score between group A and group B at different time points

Table 3 reveals that there was no statistically significant difference in the mean gingival index between groups A and B on day 0. However, there was a statistically significant difference in the mean gingival index on days 30, 90, and 180 between the two groups. The mean reduction in gingival index scores and the percentage of reduction were also statistically significant between days 0 and 30, days 0 and 90, and between days 0 and 180 [Graph 2].

**Table 3:** Gingival index scores in group A and group B at different time points

Variable	Group A (n = 30) mean ± SD	Group B (n = 30) mean ± SD	*P* value
Gingival index			
Day - 0	1.16 ± 0.30	1.21 ± 0.34	0.55 (ns)
Day - 30	0.93 ± 0.23	0.49 ± 0.46	<0.0001(sig)
Day - 90	0.88 ± 0.42	0.41 ± 0.41	<0.0001(sig)
Day - 180	0.78 ± 0.44	0.26 ± 0.38	<0.0001(sig)
Change			
Day 0 - 30	0.23 ± 0.35	0.72 ± 0.55	<0.0001(sig)
Day 0 - 90	0.28 ± 0.50	0.80 ± 0.41	<0.0001(sig)
Day 0 - 180	0.38 ± 0.49	0.95 ± 0.49	<0.0001(sig)
Percentage change from day - 0 to			
Day - 30	17.0 ± 25.5	57.5 ± 42.4	<0.0001(sig)
Day - 90	20.4 ± 43.3	67.7 ± 33.7	<0.0001(sig)
Day - 180	30.2 ± 43.2	78.0 ± 35.1	<0.0001(sig)

Mean gingival index score in group A and group B on day 0 was not statistically significant (*P* = 0.55). On days 30, 90, and 180, mean gingival index in both groups was statistically significant (*P* <0.0001)

Table 4 shows that on day 0, there was bleeding on probing of all sites of Groups A and B. On day 30, there was a 60% reduction in bleeding from the sites in group A and 80% reduction in bleeding from sites in group B. On day 90, there was a 63.4% reduction in bleeding of the sites in group A and 83.3% reduction in bleeding from sites in group B. On day 180, there was a 66.7% reduction in bleeding from sites in group A and 90% reduction in bleeding from sites in group B (*P* < 0.0001). The reduction in bleeding on probing was significant in both groups A and B. The reduction in group B was significantly higher than the reduction seen in group A on the 30^th^ day, 90^th^ day, and 180^th^ day.

**Table 4:** Distribution of gingival bleeding sites for group A and group B at different time points

Time point	Category	Group A (n = 30) N%	Group B (n = 30) N%	*P* value
Day - 0	Positive	30 (100)	30 (100)	-
	Negative	0 (0)	0 (0)	
Day - 30	Positive	12 (40)	6 (20)	0.01 (sig)
	Negative	18 (60)	24 (80)	
Day - 90	Positive	11 (36.6)	5 (16.7)	< 0.0001 (sig)
	Negative	19 (63.4)	25 (83.3)	
Day - 180	Positive	10 (33.3)	3 (10)	< 0.0001 (sig)
	Negative	20 (66.7)	27 (90)	

The reduction in bleeding on probing was significant in both group A and group B. The reduction in group B was significantly higher than that of group A on 30^th^ day, 90^th^ day, 180^th^ day

Table 5 reveals that there was no statistically significant difference in the mean probing pocket depth between the two groups on day 0. On days 90 and 180, the difference in the mean probing pocket depth was statistically significant between the two groups (*P* < 0.0001). The mean reduction in probing pocket depth between days 0 to 90 and days 0 to 180 was statistically significant between the two groups (*P* < 0.0001).

**Table 5:** Probing pocket depth in group A and group B at different time points

Variable	Group A (*n* = 30)	Group B (*n* = 30)	*P* value
			
	mean ± SD	mean ± SD	
Probing pocket depth			
Day - 0	5.57 ± 0.94	5.33 ± 0.66	0.27 (Ns)
Day - 90	5.33 ± 0.96	3.60 ± 0.77	< 0.0001 (sig)
Day - 180	5.20 ± 0.88	3.67 ± 0.76	< 0.0001 (sig)
Change			
Day 0 - 90	0.24 ± 1.07	1.73 ± 0.87	< 0.0001 (sig)
Day 0 - 180	0.37 ± 1.08	1.66 ± 0.96	< 0.0001 (sig)

Mean probing pocket depth in group A and group B on Day 0 was not statistically significant (*P* = 0.27). On Days 90 and 180, mean probing pocket depth difference for both groups was statistically significant (*P* < 0.0001). There was a significant difference between the two groups in the percentage of reduction in probing pocket depth (*P* < 0.0001) from Day 0-Day 90 and Days 0 to 180

## DISCUSSION

In our study, no statistically significant difference was observed between the two groups on the 30^th^ day from the baseline, but there was a significant difference in the plaque index on the 90^th^ and 180^th^ days between the two groups [Table 1]. These findings are in consistent with the those of the earlier studies done by Vansteenberghe *et al*.,[10] Mullur *et al*.,[11] Saito *et al*.,[12] Jones *et al*.,[13] Timmerman *et al*.,[14] Hagiwara *et al*.,[15] and Vansteenberghe *et al*.[16]

In our study, the mean reduction in plaque index scores between days 0 and 30 for groups A and B was not statistically significant [Graph 1]. However, the mean reduction in plaque index scores between day 0 and day 90 and between day 0 and day 180 for Group A and Group B was statistically significant [Table 2]. The mean reduction in gingival index scores between days 0 and 30, between days 0 and 90, and between days 0 and 180 for Groups A and B were all statistically significant [Table 3]. Thus, when the gingival status was compared between Group A and Group B, a significant improvement was observed on day 30, day 90, and day 180 in the test sites [Graph 2]. This is consistent with the findings of the studies conducted by Muller *et al*.,[17] Vansteenberghe *et al*.,[10] Saito *et al*.,[12] Jones *et al*.,[13] Timmerman *et al*.,[14] Radvar *et al.*,[18] Hagiwara *et al*.,[15] Vansteenberghe *et al*.,[16] and Kinane *et al*.[19]

When group A and group B were compared, a reduction in bleeding was seen from the sites in both the groups, but the percentage of reduction in bleeding from the sites was more in group B compared to sites of group A [Table 4]. This was statistically significant and consistent with the findings of Vansteenberghe *et al*.,[16] Vansteenberghe *et al*.,[10] by Mullur *et al*.,[11] and Timmerman *et al*.[14]

A significant reduction in probing pocket depth was found in group B when compared with group A [Table 5]. This was in accordance with the studies conducted by Mullur *et al*.,[11] Vansteenberghe *et al*.,[10] Saito *et al*.,[12] Jones *et al*.,[13] Timmerman *et al*.,[14] Radvar *et al*.,[18] Makoto Umeda *et al*.,[20] Hey-Riyeom,[21] Hagiwara *et al*.,[15] Vansteenberghe *et al*.,[16] Kinane *et al*.,[19] Williams *et al*.,[3] Dean *et al*.,[22] and Greenstein *et al*.[23]

The above results show that scaling and root planing plus Minocycline microspheres provide significantly greater probing depth reduction than scaling and root planing alone. This significant change in all the clinical parameters examined in the test group, is because Arestin™ releases therapeutic doses of the drug for more than 14 days, well above the minimum inhibitory concentration needed to kill most putative pathogens for periodontal disease.[24] A pharmacokinetic study of minocycline also revealed mean dose saliva levels approximately 1,000 times higher than those in serum. This finding suggests that minocycline has minimal absorption through the periodontal pocket into serum and stays concentrated in saliva. In addition, levels of minocycline were found in saliva for longer than 14 days, suggesting a sustained release of minocycline from the local delivery system.[25] It may be concluded that Arestin™ (1 mg Minocycline microspheres) delivered in a biodegradable delivery system, proves to be an effective means of reducing the clinical signs of chronic periodontitis.

## CONCLUSION

From the results of the study, the following conclusions can be drawn:

Test sites where Minocycline microspheres were employed, displayed a statistically significant reduction in all the clinical parameters (Plaque index, Gingival index, Gingival bleeding index, Probing pocket depth) after treatment as compared to control sites, which showed only minimal changes.

A degradable, subgingivally placed drug delivery system containing 1 mg Minocycline microspheres, is a safe and efficient adjunct to scaling and root planing in the treatment of chronic periodontitis.

The results of this study confirm that Minocycline microspheres are a safe and efficient adjunct to scaling and root planing, and can produce significant clinical benefits when compared to scaling and root planing alone.
